# Photon-Counting Computed Tomography (PC-CT) of the spine: impact on diagnostic confidence and radiation dose

**DOI:** 10.1007/s00330-023-09511-5

**Published:** 2023-03-09

**Authors:** Alexander Rau, Jakob Straehle, Thomas Stein, Thierno Diallo, Stephan Rau, Sebastian Faby, Konstantin Nikolaou, Stefan O. Schoenberg, Daniel Overhoff, Jürgen Beck, Horst Urbach, Jan-Helge Klingler, Fabian Bamberg, Jakob Weiss

**Affiliations:** 1 Department of Diagnostic and Interventional Radiology, University Hospital, Hugstetter Straße 55, 79106 Freiburg, Germany; 2 Department of Neuroradiology, University Hospital, Breisacher Straße 64, 79106 Freiburg, Germany; 3 Department of Neurosurgery, University Hospital, Breisacher Straße 64, 79106 Freiburg, Germany; 4 Siemens Healthcare GmbH, Forchheim, Germany; 5 Department of Diagnostic and Interventional Radiology, University Tuebingen, Hoppe-Seyler Straße 3, 72076 Tuebingen, Germany; 6 Department of Radiology and Nuclear Medicine, University Medical Center Mannheim, Heidelberg University, Theodor-Kutzer-Ufer 1-3, 68167 Mannheim, Germany

**Keywords:** Tomography, X-ray computed, Radiation dosage, Artifacts, Bone screws, Radiology

## Abstract

**Objectives:**

Computed tomography (CT) is employed to evaluate surgical outcome after spinal interventions. Here, we investigate the potential of multispectral photon-counting computed tomography (PC-CT) on image quality, diagnostic confidence, and radiation dose compared to an energy-integrating CT (EID-CT).

**Methods:**

In this prospective study, 32 patients underwent PC-CT of the spine. Data was reconstructed in two ways: (1) standard bone kernel with 65-keV (PC-CT_std_) and (2) 130-keV monoenergetic images (PC-CT_130 keV_). Prior EID-CT was available for 17 patients; for the remaining 15, an age–, sex–, and body mass index–matched EID-CT cohort was identified. Image quality (5-point Likert scales on overall, sharpness, artifacts, noise, diagnostic confidence) of PC-CT_std_ and EID-CT was assessed by four radiologists independently. If metallic implants were present (*n* = 10), PC-CT_std_ and PC-CT_130 keV_ images were again assessed by 5-point Likert scales by the same radiologists. Hounsfield units (HU) were measured within metallic artifact and compared between PC-CT_std_ and PC-CT_130 keV_. Finally, the radiation dose (CTDI_vol_) was evaluated.

**Results:**

Sharpness was rated significantly higher (*p* = 0.009) and noise significantly lower (*p* < 0.001) in PC-CTstd vs. EID-CT. In the subset of patients with metallic implants, reading scores for PC-CT_130 keV_ revealed superior ratings vs. PC-CT_std_ for image quality, artifacts, noise, and diagnostic confidence (all *p* < 0.001) accompanied by a significant increase of HU values within the artifact (*p* < 0.001). Radiation dose was significantly lower for PC-CT vs. EID-CT (mean CTDI_vol_: 8.83 vs. 15.7 mGy; *p* < 0.001).

**Conclusions:**

PC-CT of the spine with high-kiloelectronvolt reconstructions provides sharper images, higher diagnostic confidence, and lower radiation dose in patients with metallic implants.

**Key Points:**

• *Compared to energy-integrating CT, photon-counting CT of the spine had significantly higher sharpness and lower image noise while radiation dose was reduced by 45%.*

• *In patients with metallic implants, virtual monochromatic photon-counting images at 130 keV were superior to standard reconstruction at 65 keV in terms of image quality, artifacts, noise, and diagnostic confidence.*

## Introduction

Surgical treatment of the spine is recommended as first-line therapy for various conditions including unstable fractures and neoplastic diseases as well as severe degeneration and is expected to further increase due to demographic changes of the population [1]. Frequently, the use of metallic implants is necessary for successful treatment [2]. Computed tomography (CT) is the imaging modality of choice to assess post-surgical outcome and potential complications of both the implant and the adjacent anatomy [2]. However, artifacts caused by the implants constitute a major challenge for accurate assessment. These artifacts have a characteristic, streak-like appearance and are caused by various physical processes, such as photon scattering, photon starvation, and beam hardening [3], with the degree of these different physical processes depending on the type and density of the material. As a result, the shift in the x-ray energy spectrum and the lack of photons arriving at the detector can lead to a substantially impaired image quality including reduced soft tissue contrast, increased image noise, or an entire signal loss posing the risk of missing relevant findings [4].

To overcome these limitations, various approaches to reduce metallic artifacts have been developed and implemented in clinical routine. The most widely used techniques comprise calculation of virtual monochromatic images at high kiloelectronvolt levels from dual-energy CT data [5, 6] or the use of dedicated metal artifact reduction (MAR) algorithms based on sinogram inpainting, frequency-split techniques, iterative reconstruction approaches, and artificial intelligence to effectively reduce artifacts [7–12].

With the recent introduction of photon-counting CT (PC-CT) into clinical routine, a novel technology for data acquisition and reconstruction has become available with the potential to fundamentally change current workflows [13]. PC-CT allows for acquiring multispectral image information in every scan, which can be binned at specific kiloelectronvolt ranges and used for further post-processing and quantitative image analysis [13]. Although the full potential of this technique is still in its infancy, exploiting the multispectral data may be helpful to reduce artifacts from metallic implants and improve diagnostic confidence at a low radiation dose [14–16].

As mainly preclinical phantom-study data are available, we aimed to investigate the clinical impact of PC-CT compared to conventional energy-integrating CT (EID-CT) for spinal imaging with respect to image quality, diagnostic confidence, and radiation dose. We hypothesize that multispectral PC-CT data with reconstruction of high-kiloelectronvolt images allow for improved diagnostic confidence at reduced radiation dose compared to the currently established clinical standard.

## Materials and methods

### Ethics approval

In this prospective study approved by the Institutional Review Board (Ethics Committee, University of Freiburg), written informed consent was obtained from all patients. All procedures were in accordance with the ethical standards of the institutional and/or national research committee and with the 1964 Helsinki Declaration and its later amendments or comparable ethical standards.

### Patient cohort

We prospectively enrolled patients with clinically indicated non-enhanced PC-CT of the spine between November 2021 and March 2022. An EID-CT of the same scan region served as reference standard, which was either available from prior CT imaging of the same patient or as a matched case with regard to age, sex and body mass index (BMI). BMI was chosen as a matching parameter as it is related to the bone density [17] which itself might influence the subjective image impression.

### CT imaging acquisition and reconstruction

#### PC-CT

All PC-CT examinations were performed on a NAEOTOM Alpha (Siemens Healthineers) in supine position and without additional contrast agent. Acquisition parameters were as follows: multispectral QuantumPlus mode, CARE Dose4D & CARE kiloelectronvolts set to manual kilovolts, tube voltage 120 kV, CARE kiloelectronvolt IQ level 85 (corresponding to effective mAs 60), rotation time 0.5 s, pitch 0.8, focal spot 0.8 × 1.2 mm, and standard collimation of 144 × 0.4 mm).

From the acquired multispectral data, a standard series (PC-CT_std_) was reconstructed at 65 keV using a dedicated bone kernel (Br64) (slice thickness of 2 mm, an increment of 2 mm) and quantum iterative reconstruction (QIR) with strength 2. These protocol settings were determined in an evaluation period preceding the current study to model the clinical standard EID-CT protocol and maintain image impression at reduced radiation dose.

In addition, a second series of virtual monoenergetic images at 130 keV was reconstructed inline exploiting the multispectral PC-CT data (PC-CT_130 keV_). All other reconstruction parameters were kept similar.

#### EID

EID-CT examinations were performed on a third-generation CT SOMATOM Definition AS (Siemens Healthineers). Images were acquired using the standard clinical protocol with single source with CARE Dose4D & CARE kiloelectronvolts set to manual kilovolts, tube voltage 120 kV, rotation time 1.0 s, pitch 0.8, focal spot 0.9 × 1.2 mm, and standard collimation of 64 × 0.6 mm. Image data was reconstructed with a Br62 standard kernel with 2 mm slice thickness, 2 mm increment, and iterative reconstruction strength 3.

### Qualitative image analysis

**Fig. 1:**
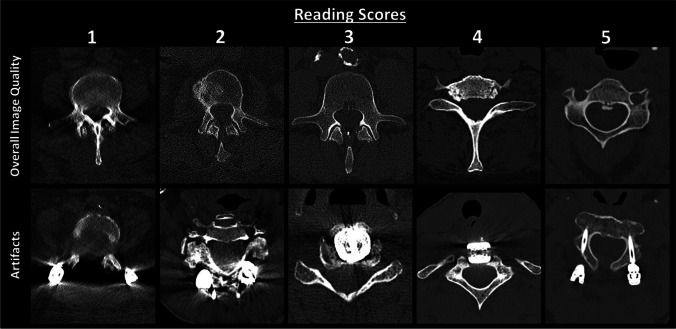
Qualitative image analysis is based on 5-point Likert scales (1 = non-diagnostic, 2 = poor image quality/severe artifacts, 3 = moderate image quality/artifacts, 4 = fair image quality/minor artifacts, 5 = excellent image quality/no artifacts). The upper row illustrates example cases for overall image quality, and the lower row illustrates example cases for artifacts

In a first reading session, image quality of PC-CT_std_ and EID-CT was assessed by four radiologists (S.R., 2 years experience; A.R., 4 years experience; J.W., 6 years experience; T.D., 8 years experience) independently in a random manner and blinded to the scanner type for (1) overall image quality, (2) edge sharpness, (3) artifacts, (4) subjective image noise, and (5) diagnostic confidence on a 5-point Likert scale (1 = non-diagnostic, 2 = poor image quality/severe artifacts, 3 = moderate image quality/artifacts, 4 = fair image quality/minor artifacts, 5 = excellent image quality/no artifacts; see Fig. 1).

All reading sessions were performed on a dedicated workstation using the publicly available post-processing platform NORA (www.nora-imaging.com). Image data were provided to the readers in 2-mm reconstructions in axial, coronal, and sagittal orientations as well as in axial thin-slice image data (0.6 mm for PC-CT and 0.75 mm for EID-CT).

In a subsequent reading session, PC-CT_std_ and PC-CT_130 keV_ images were evaluated by the same four radiologists in the subset of patients with metallic spinal implants in a similar fashion and blinded to the type of reconstruction.

### Quantitative image analysis

**Fig. 2:**
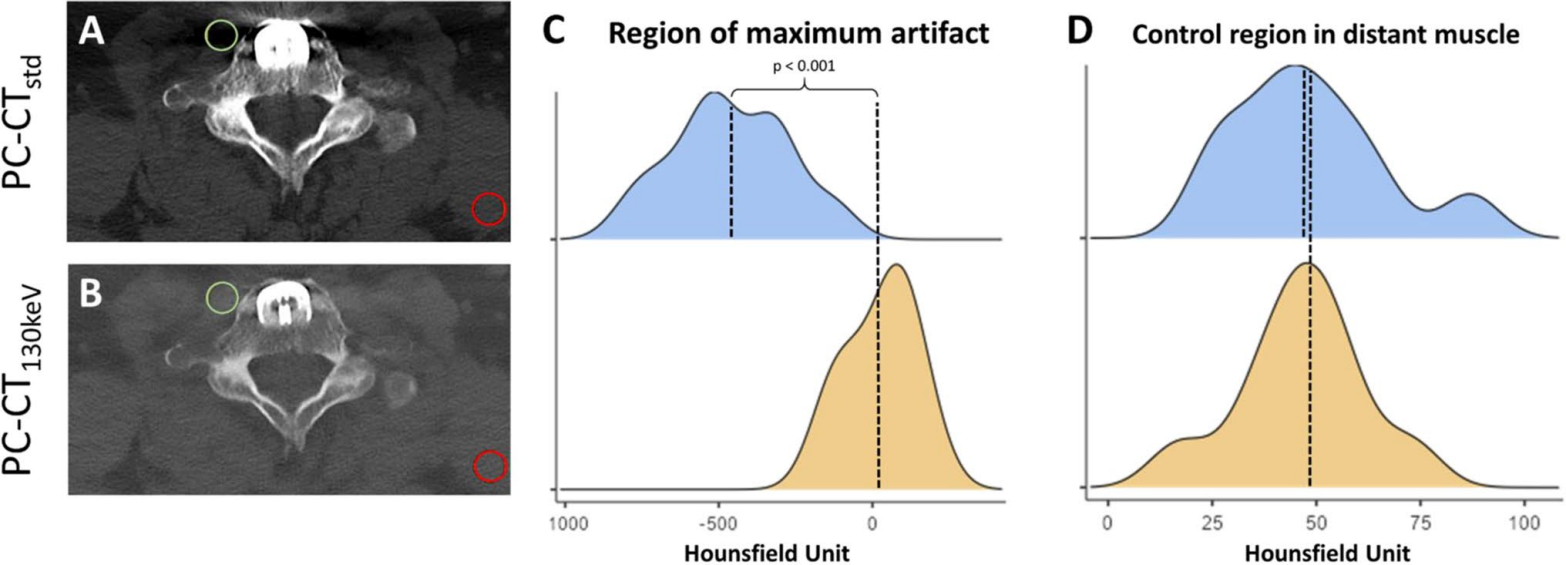
**A** and** B** Example for quantitative assessment of artifacts/artifact reduction in an area with subjectively the most severe artifact appearance (green ROI) and in a distant muscle (red ROI) not affected by the artifact serving as reference in (**A**) PC-CT_std_ and in (**B**) PC-CT_130 keV_ reconstructions. **C** and **D** Mean HU values from the ROIs of all patients were compared between PC-CT_std_ and PC-CT_130 keV_ in the artifact as well as in distant muscle. PC-CT_std_ = photon-counting CT; PT-CT_130 keV_ = PC-CT with monoenergetic reconstructions at 130 keV; HU = Hounsfield unit

Region-of-interest (ROI) measurements were performed to objectify the effectiveness of PC-CT_130 keV_ for metal artifact reduction compared to the PC-CT_std_ reconstruction. For this purpose, a 10-mm^2^ circular ROI was placed in the area with the subjectively most severe artifact appearance caused by photon starvation in PC-CT_std_. ROI size and location were kept similar for the corresponding measurements in the PC-CT_std_ reconstruction. In addition, a second ROI (10 mm^2^) was placed in a distant muscle as far away as possible from the implant and in an area subjectly not affected by the artifact serving as reference. An image example is given in Fig. 2(A/B). The mean Hounsfield unit (HU) values from these ROIs were read for PC-CT_std_ and PC-CT_130 keV_.

### Radiation dose

All dose calculations were performed using a commercially available dose management and reporting platform (DoseM, INFINITT Europe GmbH). Volume CT dose index (CTDI_vol_ [mGy]) and effective doses (mSv) were extracted for the entire cohort and cervical and lumbar scans separately and compared between the PC-CT and EID-CT examinations.

### Statistical analysis

All statistical analyses were performed using R statistics version 3.6.3 (R Core Team, https://www.R-project.org). We used the Shapiro–Wilk statistic to test for normal distribution. Continuous variables are reported as mean and standard deviation or median and ranges/interquartile ranges (IQR) as appropriate. Paired-sample *t* tests were conducted to compare age, radiation dose, and ROI HU values. Sex distribution between groups was tested with chi-square test. Differences in median reading scores were compared with the Wilcoxon rank sum test based on the average reading scores of the four readers per reading item. Values with an *α* level of 0.05 were considered statistically significant. *p* values are descriptive due to the exploratory study design.

## Results

### Patient characteristics

Spinal PC-CT was successfully performed in all 32 patients (mean age 58.8 ± 15.6 years; 18 [56%] male) included in this study. Among those, prior EID-CT of the spine was available for 17 patients; for the remaining 15 patients, a matched EID-CT cohort with similar baseline demographics could be identified (age, height, weight, and BMI [all *p* > 0.05]). In *n* = 10 patients, metallic implants were present. Patients with metallic implants were more likely men (67%), heavier, and older. Detailed patient characteristics are provided in Table 1, which were manually extracted from the electronic medical records.

**Table 1:** Patient demographics of the study participants and the matched EID-CT control cohort for the subset of patients without prior EID-CT

	PC-CT				EID-CT control cohort	
Variables	Entire PC-CT cohort	Subset with metallic implants	Subset with prior EID-CT	Subset without prior EID-CT		*p* for matching
*n*	32	10	17	15	15	
Diagnoses (*n*)						
Intervertebral disk prolapse	47% (15)	40% (4)	41% (7)	53% (8)	53% (8)	
Spondylolisthesis	16% (5)	10% (1)	24% (4)	7% (1)	0% (0)	
Fracture	13% (4)	10% (1)	6% (1)	20% (3)	20% (3)	
Metastatic disease	9% (3)	0% (0)	0% (0)	20% (3)	7% (1)	
Others	16% (5)	40% (4)	29% (5)	0% (0)	20% (3)	
Age (years)	58.8 ± 15.6	60.4 ± 16.1	57.7 ± 14.8	60.1 ± 16.9	61.1 ± 17.4	*p* = 0.875
Sex (male)	56% (18)	40% (4)	41% (7)	73% (11)	73% (11)	*p* > 0.99
Weight (kg)	80.2 ± 18.0	83.5 ± 25.4	80.4 ± 19.8	79.9 ± 16.4	78.3 ± 16.1	*p* = 0.780
Height (cm)	173 ± 7.7	173 ± 8.3	171 ± 8.9	175 ± 5.8	172 ± 7.2	*p* = 0.284
BMI (kg/m^2^**)**	26.9 ± 6.0	27.9 ± 9.2	27.6 ± 7.1	26.1 ± 4.7	26.5 ± 4.9	*p* = 0.844
Scan location						
Cervical	56% (18)	60% (6)	59% (10)	53% (8)	53% (8)	
Lumbar	44% (14)	40% (4)	41% (7)	47% (7)	47% (7)	

*PC-CT* photon-counting CT, *EID-CT* energy-integrating CT, *BMI* body mass index

### Qualitative assessment of image quality

**Fig. 3:**
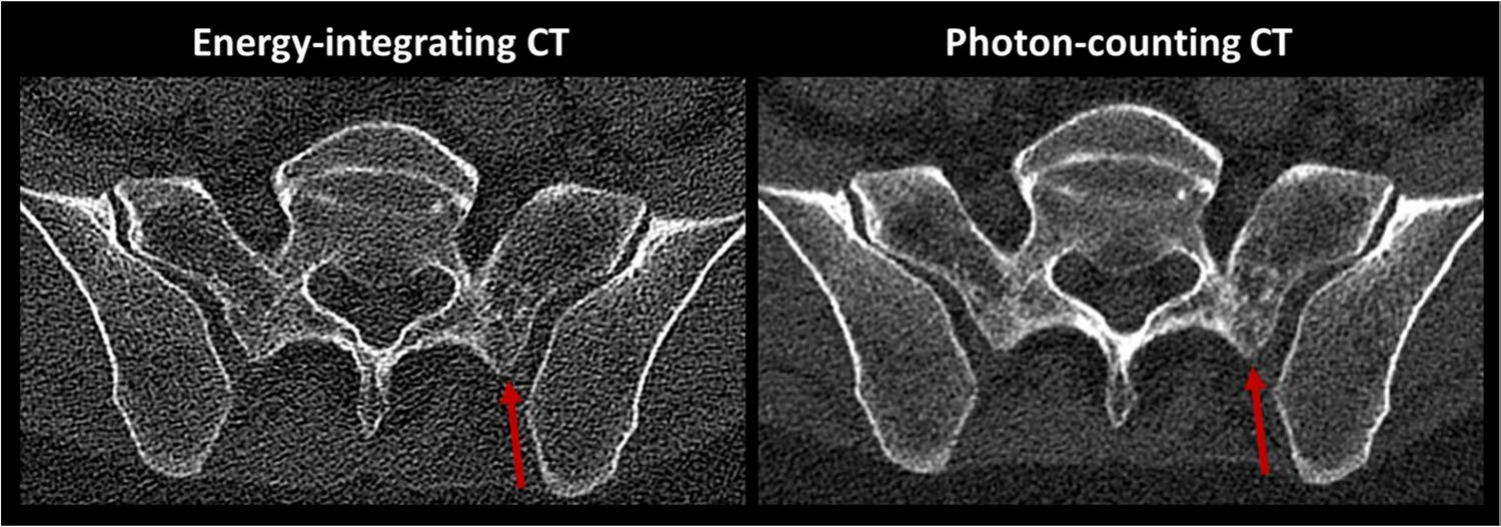
Exemplary image of a 44-year-old male patient. PC-CT shows superior delineations of the trabecular structure (red arrow) and a substantially lower image noise compared to EID-CT. Of note, the CTDI_vol_ of the EID-CT was 11.45 mGy vs. 5.56 mGy for the PC-CT examination. EID-CT = energy-integrating CT; PC-CT = photon-counting CT; CTDI_vol_ = volume computed tomography dose index

Ratings for overall image quality (3 [2–3] and 2.5 [2–3]; *p* = 0.099; PC-CT_std_ vs. EID-CT, respectively) and diagnostic confidence (3 [2–3] and 3 [1–3]; *p* = 0.364) revealed no significant differences between PC-CT_std_ and EID-CT. Similarly, no significant differences were found for artifacts (2 [1–3] and 2 [1–3]; *p* = 0.114). Sharpness was rated significantly higher (3 [2–3] vs. 2 [2–3]; *p* = 0.006) and noise significantly lower (3 [2–3] vs. 2 [1–3]; *p* < 0.001) in PC-CT_std_ compared to EID-CT (Fig. 3). None of the scans was rated non-diagnostic. An overview of all reading results is provided in Table 2.

**Table 2:** Reading scores for PC-CT_std_ versus EID-CT

	PC-CT_std_	EID-CT	*p*
	Median (IQR)	Median (IQR)	
Overall image quality	3 (2–3)	2.5 (2–3)	*p* = 0.099
Artifacts	2 (1–3)	2 (1–3)	*p* = 0.114
Diagnostic confidence	3 (2–3)	3 (1–3)	*p* = 0.364
Subjective image noise	3 (2–3)	2 (1–3)	*p* < 0.001
Edge sharpness	3 (2–3)	2 (2–3)	*p* = 0.006

Median reading scores based on the average of the four readers for the respective items

*PC-CT*_*std*_ photon-counting CT standard reconstruction, *EID-CT* energy-integrating CT, *IQR* interquartile range

**Fig. 4:**
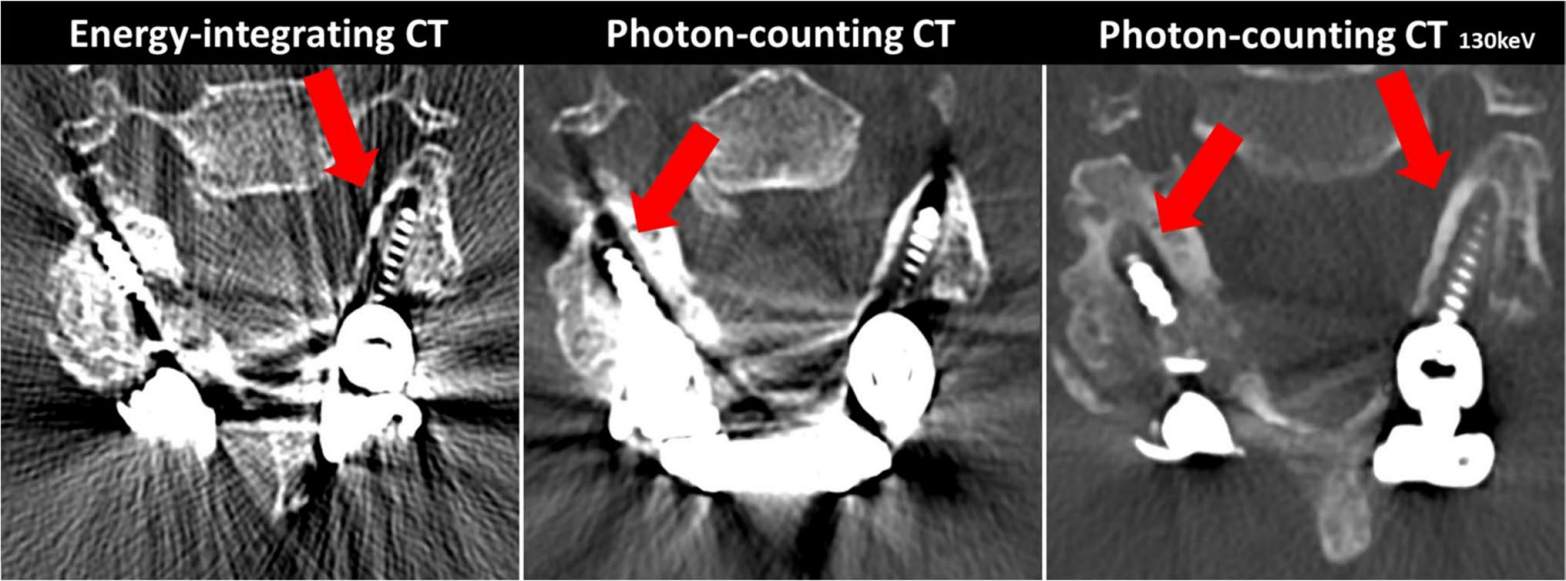
Image example of an 85-year-old patient with post-surgical CT after spinal fusion due to traumatic fracture and suspected screw loosening (window center 500 HU, window width 2000 HU). Images depict an axial slice at the height of the 5th cervical vertebra. EID-CT shows severe artifacts caused by metallic implants resulting in a substantially impaired image quality and reduced diagnostic confidence. Screw loosening was suspected in the left pedicle (arrow). After treatment of a delayed wound healing, follow-up CT imaging was performed on a PC-CT after interim external immobilization of the neck. PC-CT_std_ shows significantly reduced artifacts and better depiction of the bone resorption and also reveals loosening of the right screw (arrow). PT-CT_130 keV_ reconstruction shows almost no artifacts and clearly depicts the bone resorption around both screws (arrows). EID-CT = energy-integrating CT; PC-CT_std_ = photon-counting CT; PT-CT_130 keV_ PC-CT with monoenergetic reconstructions at 130 keV

In the subset of patients with metallic implants, reading scores for PC-CT_130 keV_ revealed significantly superior ratings vs. PC-CT_std_ for overall image quality (3 [2–4] vs. 2 [1.75–3]; *p* < 0.001), artifacts (3 [3–4] vs. 2 [1–2]; *p* < 0.001), diagnostic confidence (4 [3–4] vs. 2 [1–2.25]; *p* < 0.001), and noise (3 [2–3] vs. 2 [1–3]; *p* < 0.001), but not for sharpness (3, [2–3] vs. 3 [2–3]; *p* = 0.173) (Fig. 4). A summary of all reading scores is provided in Table 3.

**Table 3:** Reading scores for PC-CT_std_ versus PC-CT_130 keV_

	PC-CT_std_	PC-CT_130 keV_	*p*
	Median (IQR)	Median (IQR)	
Overall image quality	2 (1.75–3)	3 (2–4)	*p* < 0.001
Artifacts	2 (1–2)	3 (3–4)	*p* < 0.001
Diagnostic confidence	2 (1–2.25)	4 (3–4)	*p* < 0.001
Subjective image noise	2 (1–3)	3 (2–3)	*p* < 0.001
Edge sharpness	3 (2–3)	3 (2–3)	*p* = 0.173

Median reading scores based on the average of the four readers for the respective items

*PC-CT*_*std*_ photon-counting CT standard reconstruction, *PC-CT*_*130 keV*_ photon-counting CT monoenergetic reconstructions at 130 keV, *IQR* interquartile range

### Quantitative assessment of image quality

Quantitative measurements revealed a significant artifact reduction for PC-CT_130 keV_ compared to PC-CT_std_. Mean HU values of the ROI within the subjectively most severe artifact were significantly lower in the PC-CT_std_ vs. the PC-CT_130 keV_ reconstruction (− 462 ± 191 HU vs. 26 ± 108 HU, respectively; *p* < 0.001). No significant differences were found in the measurements of the reference ROI placed in the distant muscle subjectively not affected by the artifact (48 ± 19 HU vs. 46 ± 15 HU; *p* = 0.575; Fig. 2).

### Radiation dose

Radiation dose analyses revealed a significantly lower CTDI_vol_ for PC-CT compared to EID-CT with a mean CTDI_vol_ of 8.83 vs. 15.7 mGy; *p* < 0.001 for the entire cohort. Similar results were found when looking at the cervical and lumbar examinations separately (*p* ≤ 0.007). This observation was supported by the effective doses, which were lower for PC-CT than for EID-CT for both the entire cohort (1.64 vs. 4.68 mSv; *p* < 0.001) and the cervical (1.33 vs. 4.21 mSv; *p* < 0.001) and lumbar (2.04 vs. 5.27 mSv; *p* < 0.001) spine scans separately. A detailed overview is given in Table 4.

**Table 4:** Comparison of radiation dose between PC-CT and EID-CT

		PC-CT	EID-CT	*p*
Entire cohort (*n* = 32)	CTDIvol (mGy)	8.83 ± 3.56	15.7 ± 6.18	*p* < 0.001
	Effective dose (mSv)	1.64 ± 0.97	4.68 ± 2.51	*p* < 0.001
Cervical spine (*n* = 18)	CTDIvol (mGy)	9.29 ± 3.39	18.3 ± 4.35	*p* < 0.001
	Effective dose (mSv)	1.33 ± 0.73	4.21 ± 2.00	*p* < 0.001
Lumbar spine (*n* = 14)	CTDIvol (mGy)	7.56 ± 3.42	14.0 ± 6.39	*p* = 0.007
	Effective dose (mSv)	2.04 ± 1.11	5.27 ± 2.99	*p* = 0.001

*PC-CT* photon-counting CT, *EID-CT* energy-integrating CT, *CTDI*_*vol*_ volume CT dose index

## Discussion

In this study, we investigated the clinical impact of PC-CT compared to conventional EID-CT for spinal imaging with respect to image quality, diagnostic confidence, and radiation dose. We found that PC-CT enables a significantly improved edge sharpness and less image noise compared to the current clinical standard while radiation dose can be substantially reduced (CTDI_vol_ by 45%; effective dose by 65%). In addition, inline reconstruction of high-kiloelectronvolt monoenergetic reconstructions at virtual 130 keV provides significantly higher diagnostic confidence in patients with metallic implants while metal artifacts were effectively reduced.

These results are of clinical importance as CT imaging of the spine is routinely performed in the workup of patients with chronic back pain, trauma, and oncological diseases for both treatment planning and follow-up [18, 19]. As metallic implants are frequently necessary for successful treatment, they are encountered on a considerable number of scans [2]. Despite substantial advances in recent years, artifacts caused by such implants still pose diagnostic challenges with the risk for delayed diagnosis of implant failure and associated complications. The investigated approach using a high-kiloelectronvolt monoenergetic reconstruction has emerged as a reliable and efficient way for artifact reduction [20–22]. Laukamp et al. found that virtual monoenergetic reconstructions at 200 keV allowed for a significant artifact reduction in patients with hip replacement [23]. In addition, Hokamp et al. reported that high-kiloelectronvolt monoenergetic images can effectively reduce artifacts of dental hardware [24]. In line with these results, we found that high-kiloelectronvolt reconstructions at 130 keV significantly reduce artifacts and increase diagnostic confidence in patients with metallic spinal implants. Up till now, dedicated dual-energy CT scan modes were necessary for this approach which had to be chosen in advance of the scan. With the recent introduction of PC-CT into clinical routine, a new method has become available, which allows for acquiring the necessary information in every scan by using the multispectral properties of the new detector technology [13]. From data acquired with the QuantumPlus mode, this facilitates reconstruction of virtual monoenergetic images at arbitrary kiloelectronvolt levels between 40 and 190 keV at a significantly reduced radiation dose.

Besides virtual high-kiloelectronvolt monoenergetic reconstructions, dedicated algorithms have been proposed to effectively reduce artifacts caused by metallic implants. These algorithms have shown promising results for various types of implants such as dental hardware and hip prosthesis [25–27], especially when combined with high-kiloelectronvolt monoenergetic reconstruction [3, 25, 28, 29]. When the data for this study was collected, dedicated metal artifact reduction software was not clinically available for the investigated PC-CT system. This has changed in the meantime and will be evaluated in future work.

The new PC-CT detector technology not only enables acquisition of multispectral data but also has a substantially higher resolution compared to current state-of-the-art EID-CT systems [30, 31]. This is of particular benefit for the depiction of subtle structures such as trabecular bone [32]. Preliminary results have shown superior spatial resolution and delineation of the paranasal sinuses and temporal bone at a significantly reduced dose compared to a conventional EID-CT scanner [33, 34]. Our results also revealed a significantly higher edge sharpness of osseous structures/details in comparison to the current clinical standard while significantly reducing radiation dose. As the PC-CT protocol was set up to mimic the current scanner in terms of subjective image impression, an even higher dose reduction seems possible when fully exploiting the new detector technology and higher dose efficiency [35].

The following limitations of this study need to be considered. First, no detailed information (such as composition or alloy) of the metallic implants was available. Thus, the impact of different implant types on image quality could not be further investigated. Second, no dedicated metal artifact reduction algorithms were investigated as they were not yet clinically available for PC-CT. Further, the sample size, especially of patients with metallic implants, was relatively small. Future prospective investigations are necessary to confirm our findings. Finally, the EID-CT scans were acquired on a scanner without a dual-source detector; thus, virtual monochromatic data was not available. Despite thorough blinding of the readers, identification of PC-CT scans vs. EID-CT scans based on image sharpness and resolution cannot be finally excluded. Lower radiation dose exposure and improved image quality can be assumed for more recent, third-generation scanners even though our comparison may more realistically represent the clinical routine given their broader availability.

In conclusion, PC-CT of the spine with high-kiloelectronvolt monoenergetic reconstructions provides significantly sharper images and higher diagnostic confidence in patients with metallic implants and enables effective artifact reduction at a low radiation dose. This is of clinical importance, as follow-up imaging is frequently performed, and early diagnosis of post-surgical complications (implant failure, screw loosening) is important to initiate appropriate interventions.

## Abbreviations

BMI: Body mass index
CT: Computed tomography
CTDIvol: Volume CT dose index
EID-CT: Energy-integrating CT
MAR: Metal artifact reduction
PC-CT: Photon-counting computed tomography
PC-CT_130 keV_: 130-KeV monoenergetic PC-CT images
PC-CT_std_: Standard bone kernel PC-CT images
QIR: Quantum iterative reconstruction
ROI: Region of interest

## Declarations

### Guarantor

The scientific guarantor of this publication is Prof. Dr. Fabian Bamberg, MD, MPH.

### Statistics and biometry

One of the authors has significant statistical expertise.

### Methodology

• prospective

• observational

• performed at one institution
